# Multi-omics analysis reveals key immunogenic signatures induced by oncolytic Zika virus infection of paediatric brain tumour cells

**DOI:** 10.1038/s41598-025-97804-8

**Published:** 2025-04-16

**Authors:** Matthew Sherwood, Thiago G. Mitsugi, Carolini Kaid, Brandon Coke, Mayana Zatz, Kevin Maringer, Oswaldo K. Okamoto, Rob M. Ewing

**Affiliations:** 1 School of Biological Sciences, Faculty of Environmental and Life Sciences, University of Southampton, B85, Life Sciences Building, University Road, Highfield, Southampton, Hants. SO17 1BJ UK; 2 Centro de Estudos do Genoma Humano e Células-Tronco, Departamento de Genética e Biologia Evolutiva, Instituto de Biociências, Universidade de São Paulo, Cidade Universitária, São Paulo, SP 05508-090 Brazil; 3 The Pirbright Institute, Ash Road, Pirbright, Surrey GU24 0NF UK

**Keywords:** Paediatric brain tumours, Zika virus, Oncolytic virotherapy, RNA-sequencing, TNF signalling, Multiplex ELISA, Cytokine, Paediatric cancer, CNS cancer, Virus-host interactions, Transcriptomics

## Abstract

Brain tumours disproportionately affect children and are the largest cause of paediatric cancer-related death. Novel therapies that engage the immune system, such as oncolytic viruses (OVs), hold great promise and are desperately needed. Zika virus (ZIKV) infects and destroys aggressive cells from multiple paediatric central nervous system (CNS) tumours. Despite this, the molecular mechanisms underpinning this response are largely unknown. We comprehensively investigate the transcriptomic response of paediatric medulloblastoma and atypical teratoid rhabdoid tumour (ATRT) cells to ZIKV infection. We observe conserved TNF signalling and cytokine signalling-related signatures and show that the TNF-alpha signalling pathway is implicated in oncolysis by reducing the viability of ZIKV-infected brain tumour cells. Our findings highlight TNF-alpha as a potential prognostic marker for oncolytic ZIKV (oZIKV) therapy. Complementing our analysis with a 49-plex ELISA, we demonstrate that ZIKV infection induces a clinically relevant and diverse pro-inflammatory brain tumour cell secretome, including TNF-alpha. We assess publicly available scRNA-Seq data to model how ZIKV-induced secretome paracrine and endocrine signalling may orchestrate the anti-tumoural immune response during oZIKV infection of brain tumours. Our findings significantly contribute to understanding the molecular mechanisms governing oZIKV infection and will help pave the way towards oZIKV therapy.

**Supplementary Information:**

The online version contains supplementary material available at 10.1038/s41598-025-97804-8.

## Introduction

Malignant paediatric CNS tumours, including medulloblastoma and ATRT, are the most common solid childhood cancer and are the leading cause of mortality in paediatric cancer patients^1^. Current therapy regimens are aggressive and frequently result in debilitating long-term sequelae that ultimately impair the quality of life of patients who do not succumb to fatal tumour recurrence. There is a clear and unmet need for less toxic and more targeted therapies, especially those capable of co-opting the immune system against the tumour. OVs selectively infect and kill cancer cells, thus minimising long-term sequelae by reducing the need for high doses of chemotherapy and radiotherapy. Their efficacy stems from a two-pronged mechanism of action where the virus can directly lyse infected cancer cells (oncolysis) and elicit an anti-tumoural immune response. Many cancers, including medulloblastoma and ATRT, contain cancer stem-like cells that drive poor prognosis factors such as elevated tumour heterogeneity, metastasis and therapeutic resistance^2,3^. The ability of OVs to target these aggressive stem-like cells is a unique advantage in overcoming the therapeutic resistance of these highly heterogeneous and malignant cancers. To date, four OVs have been approved for clinical use, and > 200 OV clinical trials are underway to treat aggressive forms of cancer^4^. OV research has gained momentum since 2015, when the first FDA approval was granted for a modified form of herpes simplex virus (HSV) type 1 (T-VEC) to treat recurrent adult melanoma^5^. In 2022, a breakthrough in the fight against brain cancer was made when the Japanese Ministry of Health, Labor and Welfare approved the oncolytic herpes virus Delytact (G47∆) for the treatment of residual or recurrent adult glioblastoma^6^. A dozen clinical trials have assessed OV therapy against paediatric brain tumours, half of which have been completed, and some have reported improved patient survival. Crucially, all report that OVs are primarily accompanied by low-grade adverse events, with only rare instances of grade 3 adverse events^7–11^.

Zika virus (Orthoflavivirus zikaense) is a positive-sense single-stranded RNA (+ ssRNA) virus in the *Orthoflavivirus* genus. Maternal ZIKV infection during the first, second and third trimesters of human pregnancy results in vertical transmission in 47%, 28% and 25% of cases, and congenital ZIKV syndrome (CZS) symptoms in 9%, 3% and 1% of cases, respectively^12^. ZIKV infects fetal radial glial (RGC) and neural precursor (NPC) cells, leading to cell death and growth reduction in the fetal brain^13–16^. Postnatal ZIKV infection can result in CNS cell infection, including neural progenitors, mature neurons, and various glial cells (astrocytes, oligodendrocytes and microglia)^17–19^. Postnatal ZIKV infection is primarily asymptomatic, with the disease generally being self-limiting and symptoms resolving within two to seven days^20^. Both ZIKV-infected children and adults suffer from the same main acute symptoms: headache, fever, rash (exanthema), joint pain (arthralgia), conjunctivitis, and muscle pain (myalgia)^21,22^. In rare instances, more severe conditions such as Guillain-Barré Syndrome, meningitis and encephalitis can occur; however, these primarily affect adults rather than children^20,23^. Work by ourselves and others has shown that ZIKV can infect and destroy aggressive cells from paediatric (medulloblastoma, ATRT, diffuse midline glioma (DMG), ependymoma and neuroblastoma) and adult (glioma and meningioma) nervous system tumours^24–31^. We demonstrated that ZIKV effectively targets and destroys human CNS tumour cells and can inhibit metastatic spread without causing neurological damage in xenograft mouse models^24,32^. ZIKV-induced tumoural immune cell infiltration has been documented to include lymphoid (CD4+ T, CD8+ T and natural killer (NK) cells) and myeloid (monocytes, macrophages, dendritic cells (DCs) and microglia) cells^33–35^. This leads to ZIKV-induced tumour clearance and long-term immunity against tumour cells in immunocompetent glioma mice models, which is dependent on CD4+ and CD8+ T cells^33–35^. In a pre-clinical study, members of our research team confirmed ZIKV efficacy against spontaneous intracranial canine tumours^36^. ZIKV infection reduced tumour volume, induced an anti-tumoral immune response, improved clinical symptoms, and did not cause any persisting adverse conditions^36^.

Large data and omics techniques have revolutionised our understanding of biological systems. As these techniques are yet to be applied to oZIKV infection, the biology governing the response in paediatric brain tumour cells is generally unknown, with WNT signalling being the only known molecular mechanism involved^24^. Assessing oZIKV infection of tumour cells alongside clinically relevant CZS patient-derived NPCs is important to tease out any common or differential mechanisms that ZIKV utilises for either tumour cell oncolysis or its fetal neuropathology. Despite this, CZS patient-derived NPCs are yet to be incorporated into oZIKV research. The efficacy of most OVs is heavily dictated by the ability to induce immunogenic cell death (ICD) and a concomitant anti-tumoural immune response^37^. It is currently unclear how intracellular or extracellular factors, such as signalling pathways or cytokine secretion, may contribute to ZIKV ICD and orchestrate the anti-tumoural immune response.

Here, we perform a temporal transcriptomic survey of ZIKV infection in two embryonal brain tumour cells we previously showed to be highly susceptible to oZIKV infection in vitro and in vivo, and two CZS patient-derived NPCs^24,32,38,39^. We observe cell type-specific responses during early ZIKV infection (12–24 hours post-infection (hpi)). NPCs are highly sensitive to ZIKV infection and undergo vast downregulation of neurodevelopmental processes and essential upstream signalling pathways, likely underpinning the development of CZS in these patients^40^. In ZIKV-infected paediatric brain tumour cells, we observe transcriptomic signatures indicative of ICD, TNF pathway and cytokine signalling responses. We show the upregulation of multiple TNF-TNFR signalling pathways following infection, and we identify TNF-alpha as a potential prognostic marker for brain tumour oZIKV therapy. Investigating cytokine signalling, we generate the most comprehensive ZIKV-infected cancer cell secretome to date and show that ZIKV infection induces a diverse and predominantly pro-inflammatory secretome from paediatric brain tumour cells. Assessing publicly available scRNA-Seq data, we employ an in silico approach to model how the ZIKV secretome may contribute to an anti-tumoural immune response. We achieve this by assessing how our identified ZIKV-induced pro-inflammatory secretome may interact with medulloblastoma TME cells via paracrine signalling or polarise lymph node immune cells via endocrine signalling. In summary, our findings shed light onto the molecular mechanisms underpinning oZIKV infection of paediatric brain tumour cells and contribute to the ultimate goal of developing an oZIKV virotherapy for paediatric cancer patients.

## Results

### ZIKV infection induces cell type-specific transcriptome responses

**Fig. 1:**
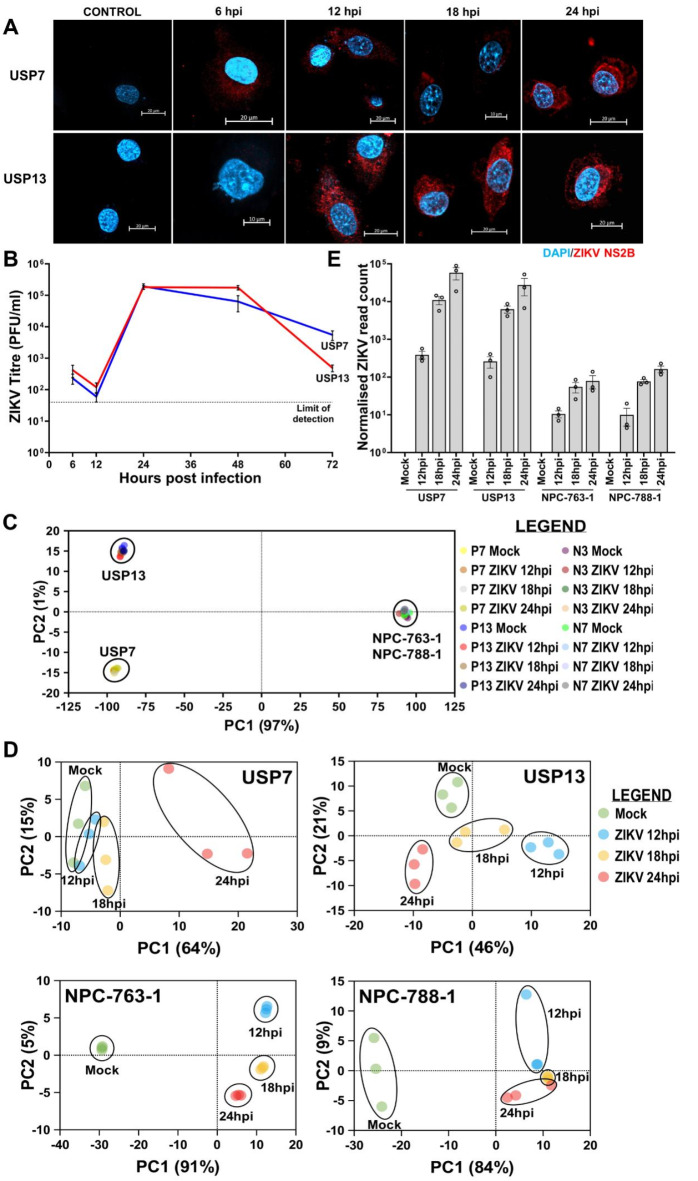
ZIKV infection dynamics in brain tumour and neural precursor cells. (**A**) Confocal microscopy of ZIKV-infected USP7 and USP13 brain tumour cells across the first 24 h of infection, staining for ZIKV replication (ZIKV NS2B, red) and the nucleus (DAPI, blue). (**B**) USP7 and USP13 cell ZIKV titre across 72 h of infection (Mean ± SEM, *N* = 4). PCA plots of (**C**) all RNA-Seq samples and (**D**) individual USP7, USP13, NPC-763-1, and NPC-788-1 cell lines. (**E**) RNA-Seq ZIKV genome count across the different infection conditions in USP7, USP13, NPC-763-1, and NPC-788-1 cells (Mean ± SEM, *N* = 3). Abbreviations, Zika virus (ZIKV), USP7 (P7), USP13 (P13), NPC-763-1 (N3), NPC-788-1 (N7), hours post-infection (hpi), adjusted p-value (padj), Principal Component (PC), Principal Component Analysis (PCA).

Following ZIKV infection, we observe distinct sub-cellular staining for ZIKV NS2B from 12 hpi in the aggressive paediatric USP7 ATRT and USP13 medulloblastoma cell lines (Fig. 1A). Viral titration primarily identifies an increase in the release of mature viral particles between 12 hpi and 24 hpi (Fig. 1B). Collectively, this indicates 12–24 hpi as a biologically relevant timeframe during early ZIKV infection in the brain tumour cells. To investigate this, we infected and performed RNA-Seq on USP7 (P7) and USP13 (P13) brain tumour cells for 12, 18, and 24 h. The NPC-763-1 (N3) and NPC-788-1 (N7) neural precursor cell lines were included as non-cancerous controls. We observed that both in the presence and absence of ZIKV infection, the brain tumour cell transcriptomes highly contrast that of the NPCs (Fig. 1C). Principal Component Analysis (PCA) and hierarchical clustering group the RNA-Seq samples by experimental condition, cell line, and disease state (Fig. 1C-D, Supplementary Fig. 1). Assessing ZIKV genome expression, we observe approximately 32, 131 and 356 times more viral genome counts in the tumour cells than in the NPCs at 12, 18 and 24 hpi, respectively (Fig. 1E). This highlights a greater propensity for ZIKV to replicate in brain tumour cells than in NPCs, possibly due to the dysregulation of antiviral responses during transformation. Despite the low accumulation of ZIKV genomes in the NPCs, thousands of differentially expressed genes (DEGs) are observed when ZIKV-infected samples are compared against Mock samples (Table 1), supporting the well-known highly sensitive nature of NPCs to ZIKV infection.

**Table 1:** Table of the differentially expressed genes (DEGs) observed following ZIKV infection in all four cell lines.

	Differentially expressed genes			
	P7	P13	N3	N7
12 hpi	446	2012	3826	1726
18 hpi	86	325	3066	2049
24 hpi	2349	3017	2644	1732

Significance was corrected for multiple testing by the Benjamini and Hochberg method for brain tumour DEGs (padj ≤ 0.05, Fold Change ≥ 0) and NPC DEGs (padj ≤ 0.05, Fold Change ≥ 1.5).

DEG, differentially expressed genes; hpi, hours post-infection.

**Fig. 2:**
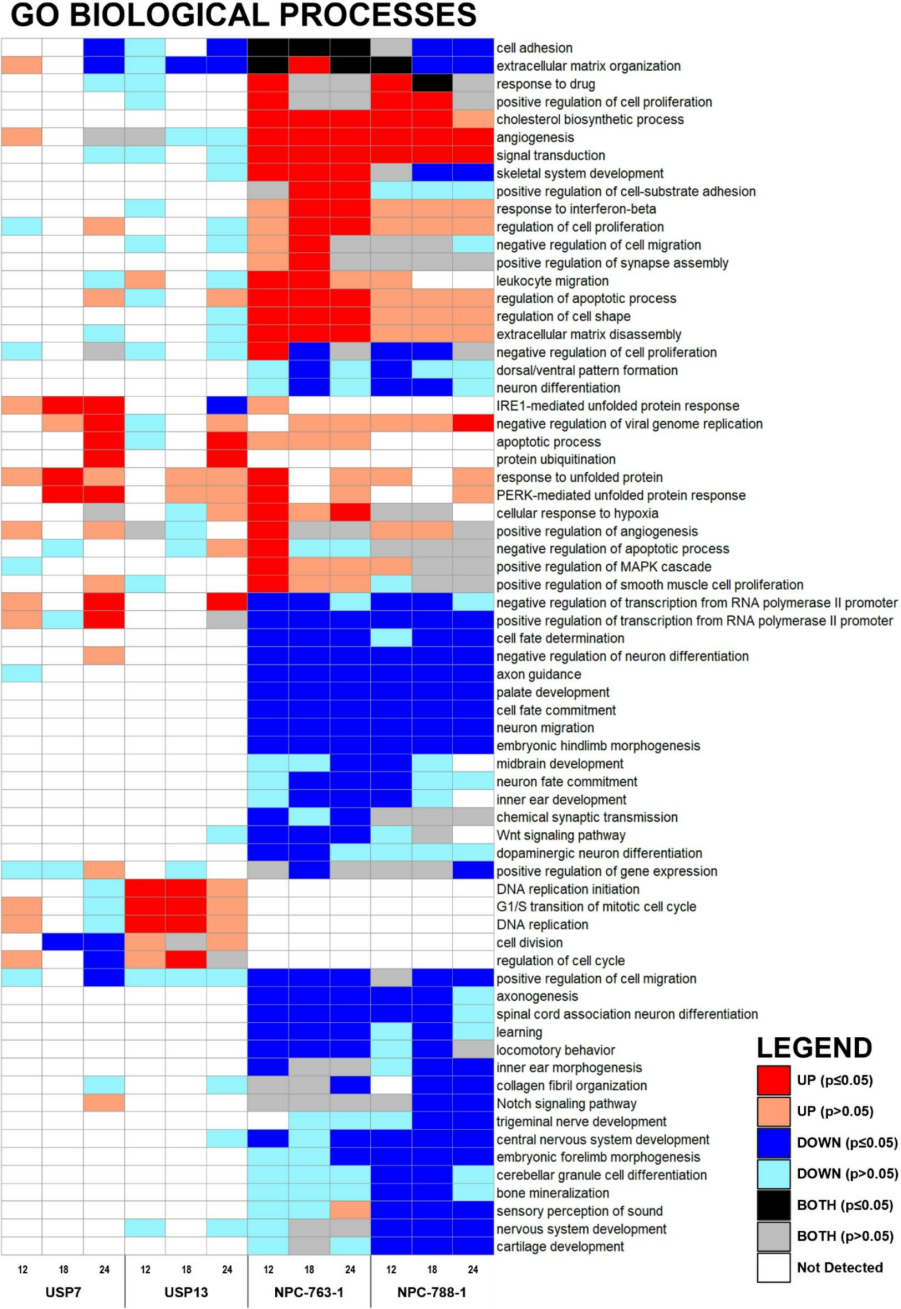
Heatmap of differentially enriched biological processes following ZIKV infection. Heatmap of the enriched Gene Ontology (GO) Biological Processes in the DEG lists at 12, 18 and 24 h of ZIKV infection in brain tumour and neural precursor cells. All terms which were significant (*p* ≤ 0.05) in at least one of the twelve infection conditions were plotted, and any non-significant (*p* > 0.05) enrichment of the given term across the remaining conditions was also plotted. If a given GO term was enriched in both the upregulated and downregulated DEG lists, then it was labelled as BOTH. Significance values are corrected for multiple testing using the Benjamini and Hochberg method (padj ≤ 0.05). Abbreviations, Zika virus (ZIKV), differentially expressed gene (DEG), USP7 (P7), USP13 (P13), NPC-763-1 (N3), NPC-788-1 (N7), Gene Ontology (GO), hours post ZIKV infection (hpi), adjusted p-value (padj).

Gene Ontology (GO) enrichment analysis identifies a multitude of differentially regulated biological processes in ZIKV-infected brain tumour and neural precursor cells (Fig. 2). We observe vast downregulation of developmental biological processes in ZIKV-infected NPCs that are known to contribute to CZS development, including terms related to morphogenesis, CNS development, neuron migration, and differentiation (Fig. 2). Multiple developmental signalling pathways which govern and coordinate these biological processes are also differentially regulated in ZIKV-infected NPCs, including WNT, Hippo, MAPK, Notch, TGF-beta, and PI3K-Akt signalling pathways (Supplementary Fig. 2). The greatest differential gene, biological process, and pathway responses in brain tumour cells occur at 24 hpi (Table 1; Fig. 2 and Supplementary Fig. 2). By 24 hpi, multiple terms implicated in cell interaction and the extracellular matrix are downregulated, whilst “apoptotic process”, “protein ubiquitination”, “protein processing in endoplasmic reticulum” and “negative regulation of transcription from RNA polymerase II promoter” terms are upregulated in both ZIKV-infected brain tumour cells (Fig. 2 and Supplementary Fig. 2). Multiple cell cycle, DNA replication and DNA repair terms are significantly upregulated specifically in USP13 cells during 12–18 hpi, whereas cell cycle-related terms are significantly downregulated in USP7 at 24 hpi; indicating that ZIKV may differentially regulate brain tumour cell growth.

**Fig. 3:**
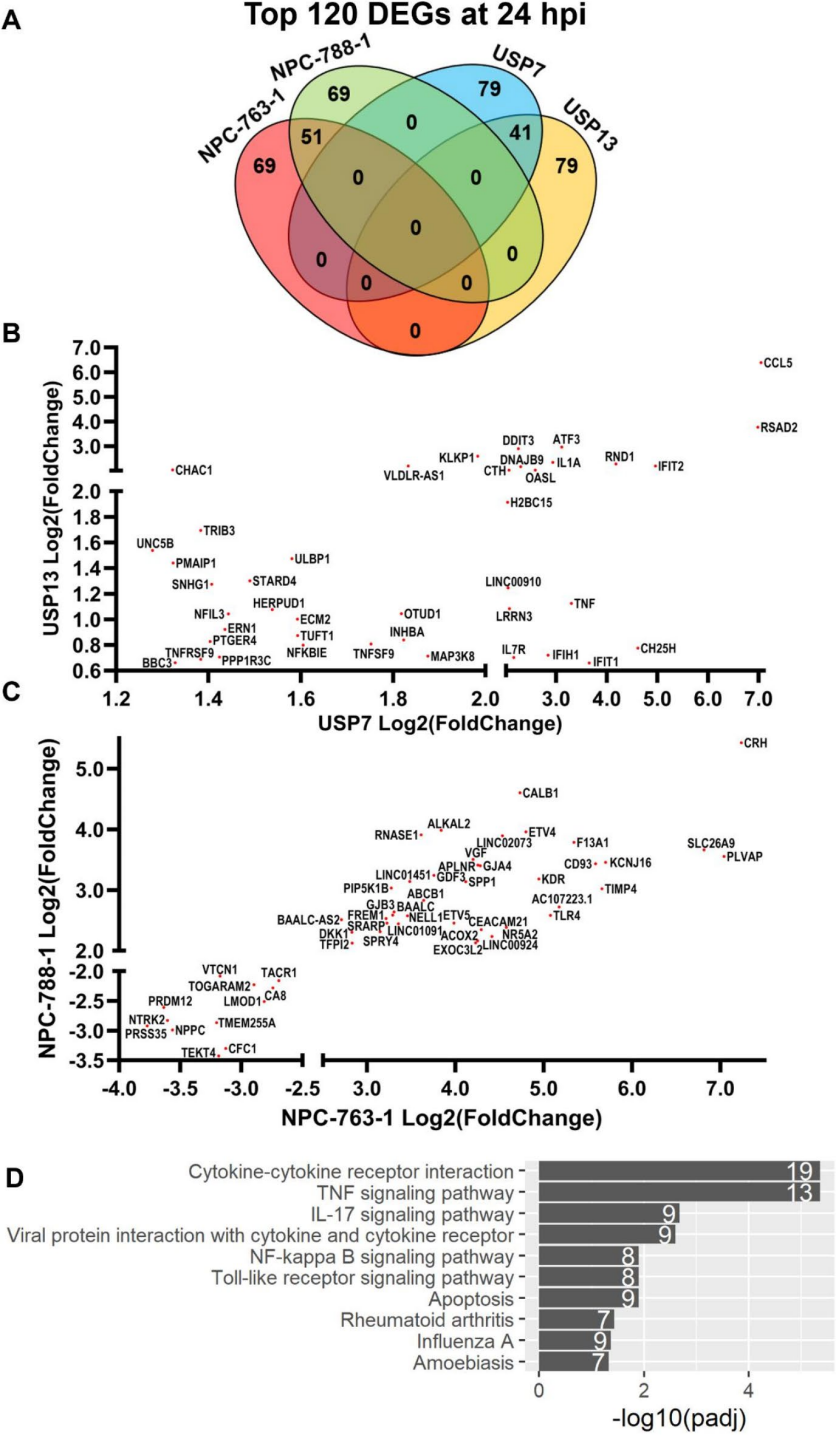
Cell type-specific responses in ZIKV-infected brain tumours and NPCs at the DEG level. (**A**) Venn diagram of the highest-ranked DEGs in each infected cell line at 24 hpi, relative to Mock. Scatter plots of RNA-Seq Log2(Fold Change) for (**B**) the 41 overlapping brain tumour DEGs and (**C**) the 51 overlapping NPC DEGs at 24 hpi, relative to Mock. (**D**) Barplot of significantly enriched KEGG pathways in the highest-ranked brain tumour DEGs at 24 hpi, relative to Mock. The white numbers at the end of each bar denote the number of DEGs identified for the given term. Significance values were corrected for multiple testing using the Benjamini and Hochberg method (padj ≤ 0.05). Abbreviations, differentially expressed gene (DEG), adjusted p-value (padj).

Comparing the highest-ranked ZIKV-induced DEGs at 24 hpi relative to Mock, we sought to identify differential molecular mechanisms that are conserved between the ZIKV-infected brain tumour cell lines. At 24 hpi, we observed a 34.2% overlap in the DEGs for brain tumour cell lines, a 42.5% overlap for NPCs, and no overlap between brain tumour cells and NPCs (Fig. 3A). Plotting the 41 shared ZIKV-infected brain tumour DEGs (Fig. 3B) and 51 shared ZIKV-infected NPC DEGs (Fig. 3C) highlights a conserved cell-type specific transcriptome response to 24 h ZIKV infection as the directionality of all DEGs plotted are the same. KEGG pathway analysis on the highest-ranked ZIKV-infected brain tumour DEGs at 24 hpi identified ten significantly enriched terms that are predominantly involved in pathogen-host interaction (“Viral protein interaction with cytokine and cytokine receptor”, “Toll-like receptor signalling pathway”, “Influenza A” and “Amoebiasis”), immune response (“Cytokine-cytokine receptor interaction”, “TNF signalling pathway”, “NF-kappa B signalling pathway” and “IL-17 signalling pathway”) and cell death (“Apoptosis”) (Fig. 3D). Considering the 41 shared ZIKV-infected brain tumour DEGs at 24 hpi (Fig. 3B), we sought to gain further insight into these enriched pathways to identify candidates for validation. We identify genes involved in TNF signalling and its downstream activity (TNF-alpha, TNFSF9, TNFRSF9, CCL5, MAP3K8, TRIB3, NFKBIE and OTUD1), the unfolded protein response (UPR) (ERN1, DDIT3, DNAJB9, ATF3 and HERPUD1), cell death (DDIT3, PMAIP1, BBC3 and CHAC1), antiviral responses (IFIT1, IFIT2, RSAD2, IFIH1 and OASL), interleukins (IL1A, IL7R and NFIL3), neuronal processes (RND1, LRRN3 and UNC5B), and lipid homeostasis (STARD4 and CH25H) (Fig. 3B). Notably, we observe joint upregulation of the UPR-regulated pro-apoptotic transcription factor DDIT3 (CHOP), its downstream pro-apoptotic gene CHAC1 and two BCL2-homologous (BH)-3-only proteins that are effectors of canonical mitochondrial apoptosis (PMAIP1 (NOXA) and BBC3 (PUMA)) (Fig. 3B). These pro-apoptotic and ER stress signatures observed at 24 hpi indicate ZIKV as a Type II ICD inducer for brain tumour cells, as expected for an ER-tropic virus. TNF and pro-inflammatory cytokine signalling are principal outcomes of ICD, and we observe terms related to these as the top two significantly enriched KEGG pathways in ZIKV-infected brain tumour cells at 24 hpi (Fig. 3D). To conclude, (i) ZIKV infection leads to conserved cell type-specific transcriptomic responses, (ii) NPCs are highly sensitive to ZIKV and undergo vast downregulation of neurodevelopmental processes following infection, and (iii) ZIKV-infected brain tumour cells portray ICD transcriptomic signatures.

### TNF-alpha is a potential prognostic marker for brain tumour oZIKV therapy

**Fig. 4:**
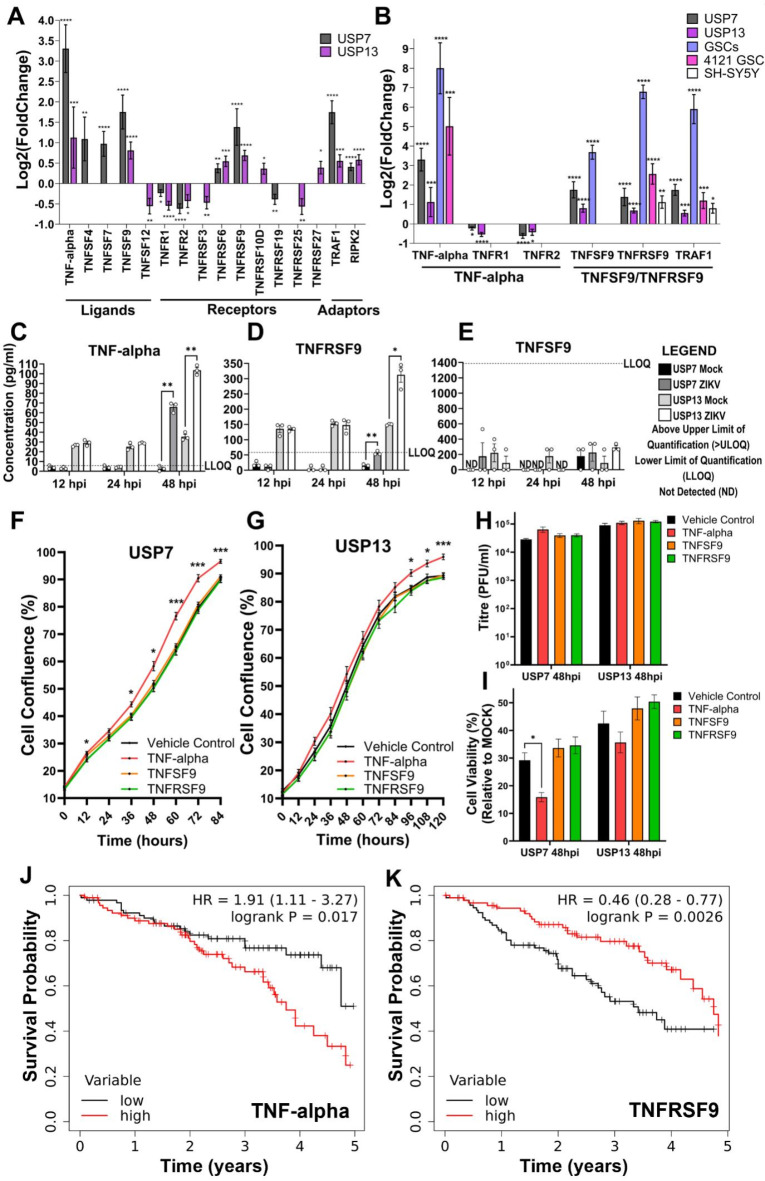
TNF signalling during ZIKV infection of brain tumour cells. (**A**) Differentially expressed TNF pathway ligands, receptors, and receptor adaptors in ZIKV-infected USP7 and USP13 brain tumour cells at 24 hpi (LFC ± lfcSE, *N* = 3). (**B**) Differential expression of principal TNF-alpha and TNFSF9/TNFRSF9 signalling pathways genes in nervous system tumour cells following ZIKV infection. ZIKV-infected RNA-Seq datasets include that generated in the current study for USP7 and USP13, and three sourced from GEO2R for GSCs, 4121 GSCs and SH-SY5Y neuroblastoma cells (LFC ± lfcSE, *N* = 3). Protein concentration of secreted (**C**) TNF-alpha, (**D**) TNFRSF9 and (**E**) TNFSF9 from USP7 and USP13 cells following 12, 24 and 48 h of ZIKV infection (Mean ± SEM, *N* = 3). (**F**) USP7 and (**G**) USP13 cell confluence following recombinant TNF protein treatment (Mean ± SEM, *n* = 8). (**H**) ZIKV titre following recombinant TNF protein treatment and ZIKV infection of USP7 and USP13 cells (Mean ± SEM, *N* = 3, *n* = 3). (**I**) USP7 and USP13 cell viability following recombinant TNF protein treatment and ZIKV infection (Mean ± SEM, *N* = 3, *n* = 3). Kaplan Meier plots to assess the correlation between medulloblastoma patient survival and the upper vs. lower quartiles of (**J**) TNF-alpha and (**K**) TNFRSF9 gene expression. Logrank P and hazard rate (HR) with 95% confidence intervals are reported. Significance values were corrected for multiple testing using the Benjamini and Hochberg method for **A,B** (padj ≤ 0.05) and **C–E** (FDR ≤ 0.05), and Dunnett’s method for **F–I** (padj ≤ 0.05). Asterisk symbol denotes level of significance: *padj ≤ 0.05; **padj ≤ 0.01; ***padj ≤ 0.001; ****padj ≤ 0.0001. Abbreviations, Zika virus (ZIKV), Glioma Stem Cell (GSC), Log2(Fold Change) (LFC), standard error of the LFC estimate (lfcSE), Above Limit of Quantification (> ULOQ), Lower Limit of Quantification (LLOQ), Not Detected (ND), hours post-infection (hpi), adjusted p-value (padj).

Assessing all differentially expressed TNF ligands, receptors, and adaptors, we observe the most highly upregulated DEGs in both ZIKV-infected USP7 and USP13 cells at 24 hpi to be key components of the TNF-alpha pathway and TNF receptor superfamily member 9 (TNFSF9/TNFRSF9) pathway (Fig. 4A). Activation of either pathway results in downstream NF-kappa B signalling, which we observe to be significantly enriched at 24 hpi in the ZIKV-infected brain tumour cells (Fig. 3D). Data mining publicly available ZIKV-infected glioma and neuroblastoma RNA-Seq datasets, we observe that TNFRSF9 and one of its adaptors (TRAF1) are significantly upregulated in all cases, TNFSF9 is significantly upregulated in three instances, and TNF-alpha is significantly upregulated in all four brain tumour datasets (Fig. 4B). Thus, these responses appear conserved across different ZIKV-infected nervous system tumour cells. Performing ProcartaPlex ELISA assays, we assessed whether the soluble TNF factors of these pathways (TNF-alpha, TNFSF9 and TNFRSF9) were secreted following infection and observed significantly increased secretion of TNF-alpha (Fig. 4C) and TNFRSF9 (Fig. 4D) from both USP7 and USP13 cells at 48 hpi. TNFSF9 was not reliably detected or quantified under any condition (Fig. 4E). Thus, we show that ZIKV infection of brain tumour cells leads to the upregulated expression and secretion of key members of both TNF alpha and TNFSF9/TNFRSF9 signalling pathways.

Next, we sought to functionally assess these soluble proteins for their (i) effect on brain tumour cell growth, (ii) anti- or pro-viral properties, and (iii) effect on brain tumour cell viability following ZIKV infection. We employed Incucyte Live Cell Imaging to analyse USP7 and USP13 cell growth by tracking cell confluence following treatment with recombinant TNF proteins (Fig. 4F and G). None of the recombinant TNF proteins were cytotoxic, and TNF-alpha significantly increased both USP7 and USP13 confluence, indicating that soluble TNF-alpha enhances brain tumour cell growth (Fig. 4F and G). Next, we assessed the effect of recombinant protein treatment on ZIKV titre (Fig. 4H) and tumour cell viability (Fig. 4I). Neither treatment with recombinant TNF-alpha, TNFSF9 nor TNFRSF9 led to an increase or decrease in viral titre, suggesting that none of the TNF proteins possess pro- or anti-viral properties, respectively (Fig. 4H). Recombinant TNF-alpha significantly reduced ZIKV-infected USP7 cell viability, and a modest but non-significant reduction was observed in ZIKV-infected USP13 cells (Fig. 4I). Performing a five-year survival analysis on clinical and gene expression data for 375 medulloblastoma patients, we observe high TNF-alpha expression to be significantly correlated with worse survival (Fig. 4J) and high TNFRSF9 expression to be significantly correlated with improved survival (Fig. 4K). Thus, TNF-alpha is a marker of poor prognosis for medulloblastoma patients, possibly by enhancing brain tumour cell growth. Here, we show that TNF-alpha (i) is a brain tumour cell growth factor, (ii) may sensitise brain tumour cells to oncolysis by augmenting ZIKV-induced reduced cell viability, and (iii) is a marker of poor prognosis for medulloblastoma. Collectively, our results indicate that TNF-alpha holds promise as a potential prognostic marker for brain tumour oZIKV therapy.

### ZIKV infection stimulates a diverse pro-inflammatory cytokine secretome from brain tumour cells

**Fig. 5:**
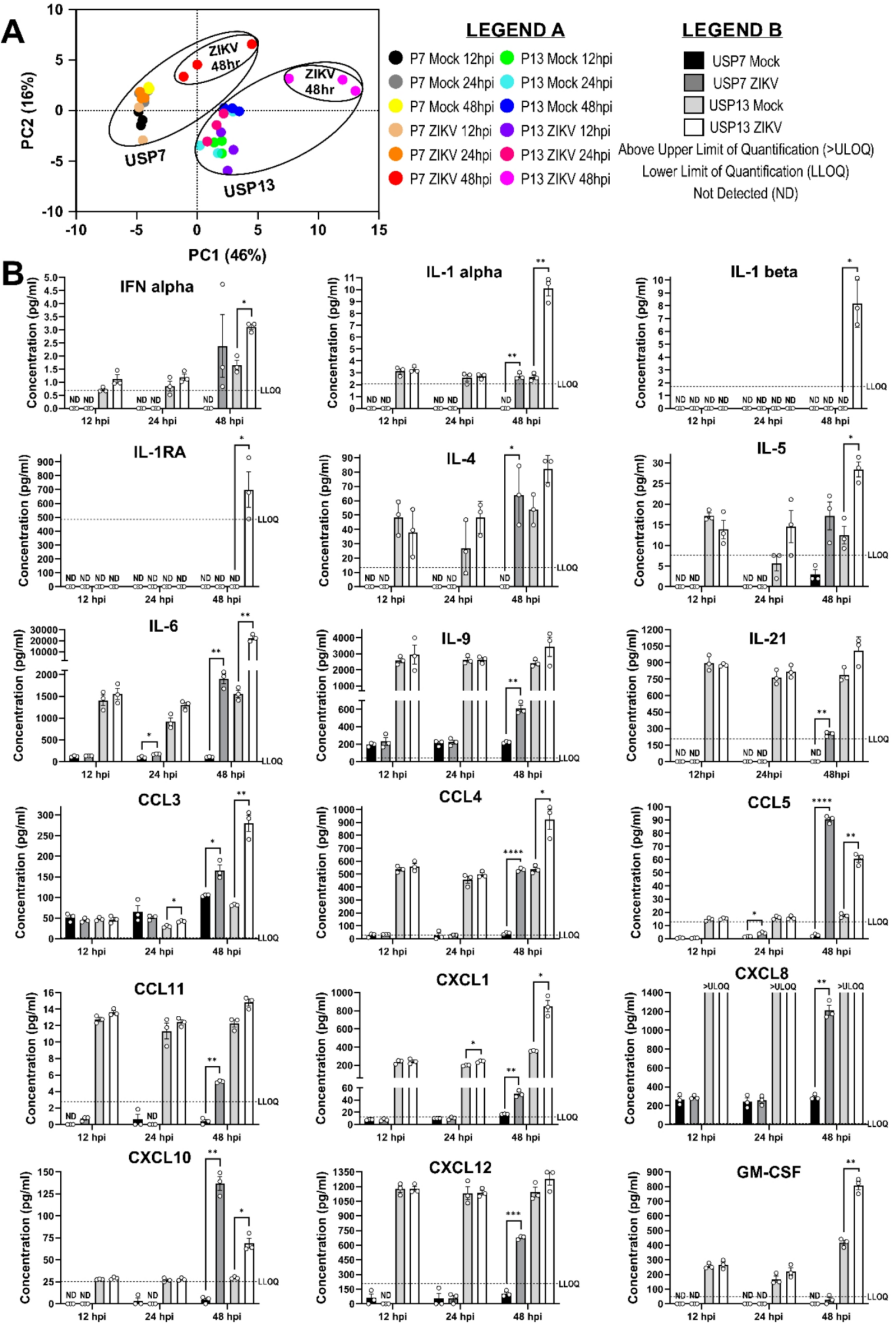
ZIKV-infected brain tumour cell secretome. (**A**) PCA plot of the 49-plex ELISA averaged Net MFI values (*N* = 3). (**B**) Differential secretion of cytokines from ZIKV-infected USP7 and USP13 cells at 12, 24 and 48 hpi, relative to Mock samples (Mean ± SEM, *N* = 3). Significance values were corrected for multiple testing using the Benjamini and Hochberg method (FDR ≤ 0.05). Asterisk symbol denotes level of significance: *padj ≤ 0.05; **padj ≤ 0.01; ***padj ≤ 0.001; ****padj ≤ 0.0001. Abbreviations, Zika virus (ZIKV), USP7 (P7), USP13 (P13), Above Limit of Quantification (> ULOQ), Lower Limit of Quantification (LLOQ), Not Detected (ND), hours post-infection (hpi), Principal Component (PC), Principal Component Analysis (PCA).

**Fig. 6:**
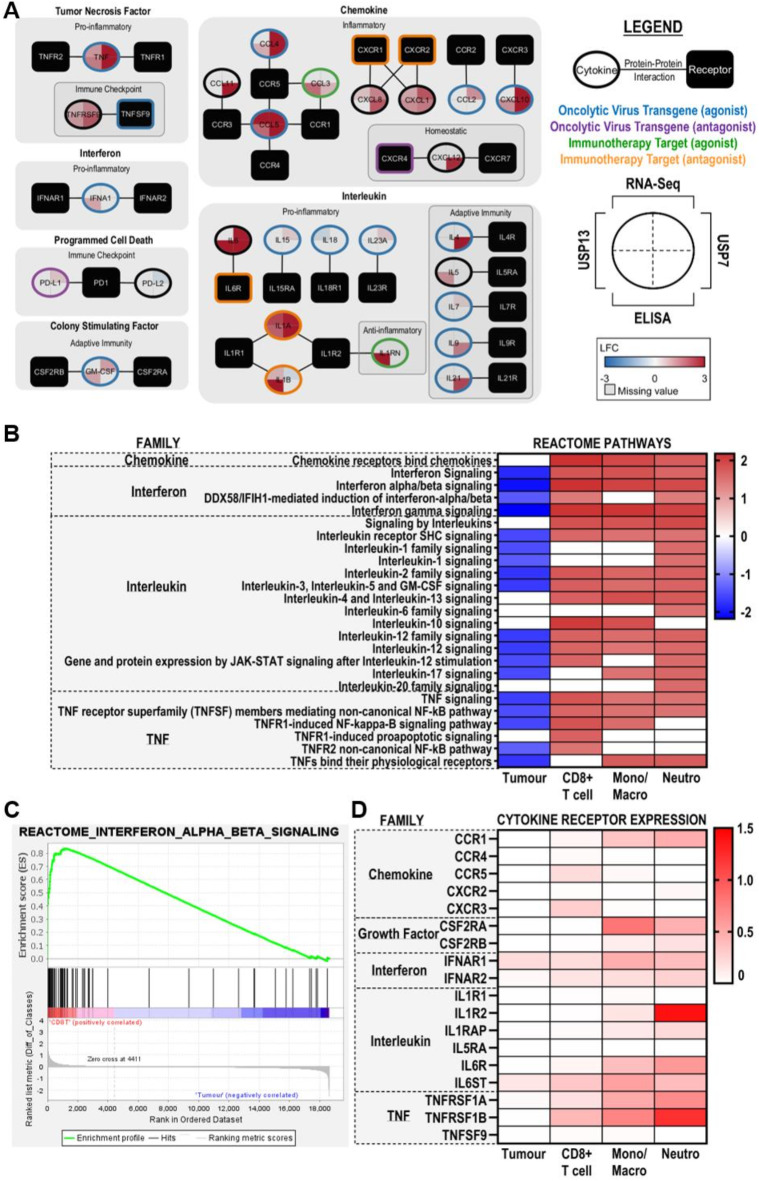
The pro-inflammatory ZIKV secretome and its predicted medulloblastoma TME paracrine signalling. (**A**) An integrated map of the 27 significantly expressed or secreted cytokines in at least one brain tumour cell line following ZIKV infection. Cytokines and their cognate receptors are clustered by their family class and sub-clustered by their primary role in the immune response. Edges denote cytokine-cytokine receptor interactions. Node shape denotes if the protein is a cytokine (oval) or receptor (rectangle). Pie chart quadrants denote the maximum LFC value observed for the given cell line (USP7 or USP13) in the given assay (RNA-Seq or ELISA). Border colour denotes if the cytokine-receptor axis is a current target of oncolytic virotherapy or immunotherapy and highlights which of the two proteins is the transgene or immunotherapy target. Heatmaps showing the (**B**) GSEA normalised enrichment score (NES) of significantly enriched cytokine-related Reactome pathways (*p* ≤ 0.05, FDR ≤ 0.25) and (**D**) cytokine receptor expression (log-transformed normalised gene count) in the four major cell types annotated in the medulloblastoma scRNA-Seq dataset. Rows for (**B**) and (**D**) are arranged by cytokine family. (**C**) Representative positively enriched cytokine-related GSEA enrichment plot (Interferon alpha/beta signalling) in immune cells (CD8+ T cells). Abbreviations, Zika virus (ZIKV), Log2(Fold Change) (LFC), Tumour microenvironment (TME), CD8+ T cells (CD8T), Monocyte/Macrophage (Mono/Macro), Neutrophil (Neutro), Gene Set Enrichment Analysis (GSEA), Normalised Enrichment Score (NES).

Extending our TNF ELISA assays to a 49-plex ELISA, we sought to validate the cytokine-related transcriptomic signatures enriched in ZIKV-infected brain tumour cells (Fig. 3D). Assessing the temporal secretion of 34 cytokines (including TNF-alpha) and 15 immune checkpoint proteins (including TNFRSF9 and TNFSF9), we generate the most comprehensive ZIKV-infected cancer cell secretome to date (Supplementary Table 1). PCA clusters the secretome by cell line and shows the 48 h ZIKV-infected condition for both cell lines to vary from all other samples (Fig. 5A). In addition to TNF-alpha (Fig. 4C), we observe significantly increased secretion of 18 cytokines, predominately at 48 hpi (Fig. 5B). Seven cytokines are significantly secreted by both infected brain tumour cells (CXCL1, IL-1 alpha, IL-6, CXCL10 (IP-10), CCL3, CCL4 and CCL5 (RANTES)), six from infected USP7 cells only (CCL11, IL-4, IL-8 (CXCL8), IL-9, IL-21 and SDF-1 alpha) and five from infected USP13 cells only (GM-CSF, IFN alpha, IL-1 beta, IL-1RA and IL-5) (Fig. 5B). Strikingly, of the 15 immune checkpoint proteins, TNFRSF9 was the only significantly secreted protein (Fig. 4D), and PD-L2 was the only other protein detected across all conditions (Supplementary Table 1). ZIKV infection did not significantly reduce the secretion of any protein. Integrating the 49-plex ELISA and RNA-Seq data, we observe 27 of the 49-plex proteins to be significantly differently expressed or secreted by USP7 or USP13 following ZIKV infection (Fig. 6A). These diverse proteins encompass chemokine, interleukin, tumor necrosis factor, interferon, colony stimulating factor and programmed cell death proteins. Considering the primary role of each protein during an immune response, we identify that ZIKV infection predominantly promotes a pro-inflammatory rather than anti-inflammatory cytokine profile (Fig. 6A). Additionally, we identify many cytokines which orchestrate adaptive immune responses. Crucially, many of these proteins are current OV transgenes or immunotherapy candidates. To conclude, ZIKV infection induces a diverse pro-inflammatory secretome from brain tumour cells, containing many clinically relevant candidates for mediating an OV-stimulated anti-tumoural immune response.

### In silico modelling predicts the medulloblastoma tumour immune microenvironment (TIME) to respond to pro-inflammatory ZIKV secretome paracrine signalling

Employing an in silico approach, we sought to model how the ZIKV-induced pro-inflammatory USP13 medulloblastoma secretome may interact with tumour and immune cells of the medulloblastoma TME. Our model predicts the indirect effects of ZIKV infection (i.e. through the ZIKV-induced brain tumour secretome) rather than the direct effects (i.e. direct interaction through ZIKV infection) on these cells. Analysing publicly available medulloblastoma scRNA-Seq, we identify positive enrichment of many cytokine-related Reactome pathways by GSEA (Fig. 6B and C) and greater cytokine receptor expression (Fig. 6D) in monocytes/macrophages, CD8+ T cells and neutrophils than in medulloblastoma tumour cells. All immune cells express receptors for TNF-alpha (TNFRSF1A and TNFRSF1B) and IFN alpha (IFNAR1 and IFNAR2) and are enriched for these pathways, indicating that all these immune cell types are primed to respond to secreted TNF-alpha and IFN alpha in the medulloblastoma TME (Figs. 6B–D). Monocytes/macrophages and neutrophils express the CCL3 and CCL5 receptor CCR1, while CD8+ T cells express the CXCL10 receptor CXCR3 and the CCL4 and CCL5 receptor CCR5. All three immune cell types are enriched for the “Chemokine receptors bind chemokines” Reactome pathway and are, therefore, likely to respond to these secreted chemokines. Additionally, monocytes/macrophages and neutrophils express the main GM-CSF receptor CSF2RA and are enriched in the “Interleukin-3, Interleukin-5 and GM-CSF signalling pathway”, indicating potential to respond to secreted GM-CSF. Finally, neutrophils express receptors for IL-1 (IL1R2 and IL1RAP) and IL-6 (IL6R and IL6ST) and are the only cell type enriched for their related pathways, indicating a possible neutrophil-specific response to secreted interleukins. Collectively, our modelling predicts that secretion of the cytokines which make up the ZIKV-induced pro-inflammatory secretome would remodel the medulloblastoma TIME.

### In silico modelling predicts the pro-inflammatory ZIKV secretome to stimulate diverse immune cell polarisations through endocrine signalling

**Fig. 7:**
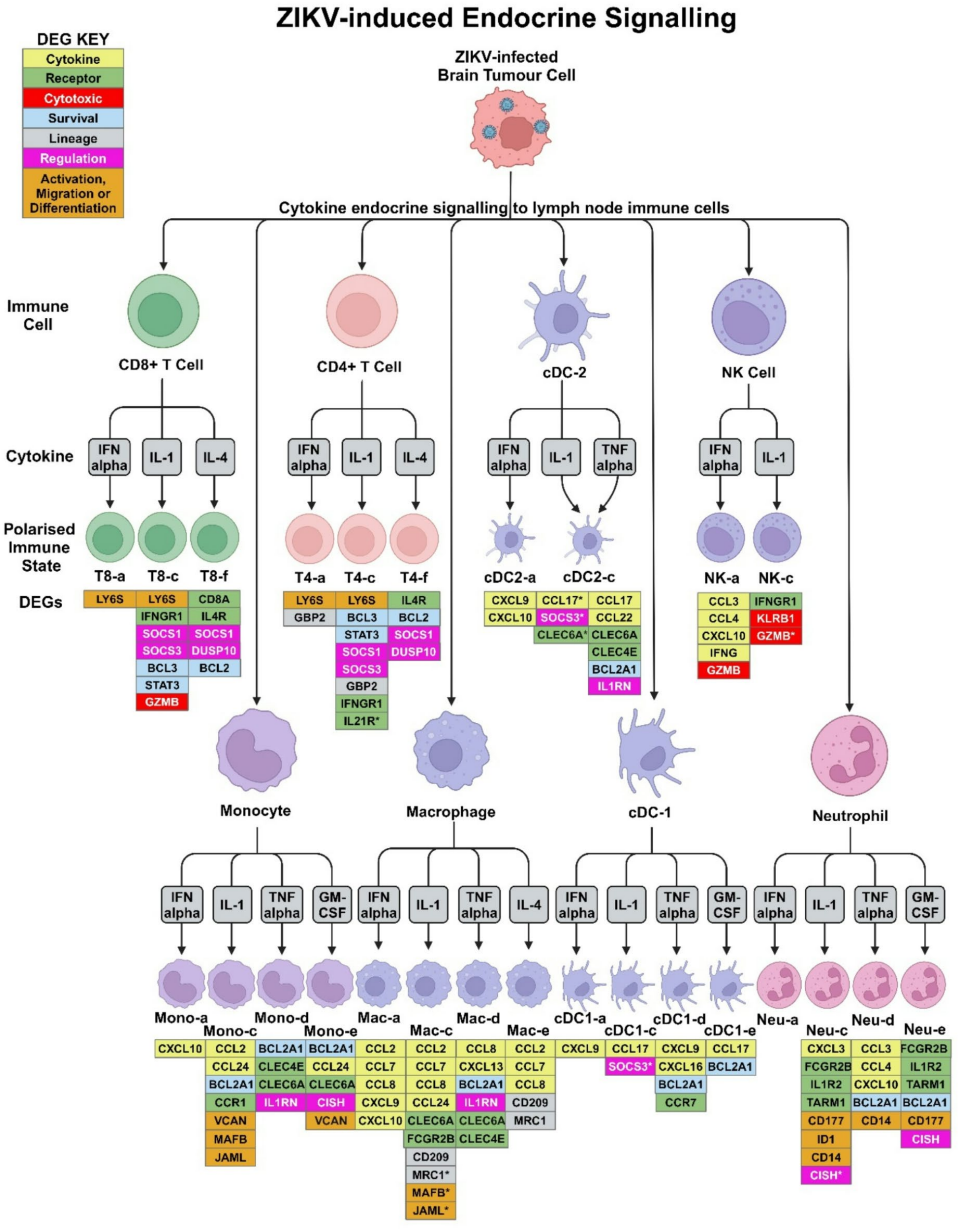
Modelling of ZIKV-induced brain tumour secretome endocrine signalling to lymph node immune cells. Diagram detailing polarisation states of lymph node immune cells following cytokine stimulation, as reported by the Immune Dictionary^41^. Only ZIKV-induced brain tumour cell-secreted cytokines that are dominant drivers of immune cell polarisation were assessed, as determined by the Immune Dictionary. Polarisation states, their nomenclature and the top 20 upregulated cytokine-induced DEGs (FDR ≤ 0.05) were sourced from the Immune Dictionary. Phenotypic DEG markers that indicate immune cell activity or phenotype following cytokine stimulation are listed below the respective polarised state, with colours denoting if the DEG is a cytokine (yellow), receptor (green), cytotoxicity-related gene (red), survival-related gene (blue), cell lineage marker (grey), cytokine signalling regulator (pink), or a gene indicating general immune cell activity (activation, migration or differentiation) (orange). IL-1 alpha and IL-1 beta induce the same polarisation state and are thus collectively represented as IL-1. * denotes a DEG induced by either IL-1 alpha or IL-1 beta, but not both. Abbreviations, Zika virus (ZIKV), differentially expressed gene (DEG), conventional Dendritic Cell (cDC), Natural Killer (NK), CD8+ T cell (T8), CD4+ T cell (T4), Monocyte (Mono), Macrophage (Macro), Neutrophil (Neu). Figure created in BioRender. BioRender.com/u63c617.

Employing an in silico approach, we next sought to model how our ZIKV-induced pro-inflammatory secretome may interact with lymph node (i.e. non-tumour-resident) immune cells, and predict if the secretome would induce immune cell polarisations towards an anti-tumoural state. To achieve this, we interrogated scRNA-Seq data in the Immune Dictionary (a dictionary documenting over 17 immune cell type responses to 86 different cytokines at the single-cell transcriptome level)^41^. Six of the 20 proteins differentially secreted by ZIKV-infected USP7 or USP13 cells (IFN alpha, IL-1 alpha, IL-1 beta, IL-4, TNF-alpha and GM-CSF) are documented in the Immune Dictionary as dominant cytokines which drive polarisation states of the main lymphoid (CD4+ T, CD8+ T and NK cells) and myeloid (monocytes, macrophages, DCs and neutrophils) cell types implicated in anti-tumoural immune responses. We sought to predict the phenotypes of these immune cells following stimulation with the six dominant cytokines by assessing (i) individual cytokine-cell interactions, (ii) the top-upregulated DEGs, and (iii) the resulting immune cell polarisation states (Fig. 7). We observe all six cytokines to be pleiotropic, polarising the eight immune cell types into 26 distinct polarization states (Fig. 7). IFN alpha, IL-1 alpha, and IL-1 beta polarise every cell type. IFN alpha induces a unique polarisation (suffix -a), and IL-1 alpha and IL-1 beta (collectively referred to as IL-1) induce a conserved polarisation (suffix -c). We observe self-regulation of cytokine signalling transduction by positive feedback receptor upregulation (e.g. IL4R), negative feedback inhibitory receptor upregulation (e.g. IL1R2), and upregulation of cytokine signalling regulators (SOCS1, SOCS3, CISH and DUSP10). Most cytokines sensitise or suppress the eight immune cell types to other cytokine signalling through upregulated expression of cytokines (e.g. CXCL9), receptors (e.g. IFNGR1), or receptor antagonists (e.g. IL1RN).

We observe TNF-alpha to act on myeloid rather than lymphoid lineage cells (Fig. 7). All TNF-alpha and GM-CSF-stimulated myeloid cells upregulate the anti-apoptotic BCL2A1 gene, indicating a BCL2A1-mediated pro-survival mechanism. TNF-alpha induces the expression of IL1RN (IL-1RA) and the pattern recognition receptors CLEC6A and CLEC4E in monocytes, macrophages and cDC2s, possibly suppressing IL-1 responses and priming myeloid cells for innate immune recognition. Upregulation of LY6S indicates CD4+ and CD8+ T cell activation by IFN alpha and IL-1 (T4-a, T4-c, T8-a and T8-c). Upregulation of the effector memory T cell marker GBP2 indicates that T4-a and T4-c likely become effector memory CD4+ T cells. Upregulation of the principal cytotoxic granzyme GZMB indicates that T8-c are likely cytotoxic CD8+ T cells. T cell survival may be driven in a BCL2-mediated manner following IL-4 stimulation (T4-f and T8-f) and a STAT3/BCL3-mediated manner following IL-1 stimulation (T4-c and T8-c). IFN alpha and IL-1 beta upregulate cytotoxic granzyme GZMB expression in NK-a and NK-c cells, and the cytotoxicity regulator KLRB1 may govern the NK-c cytotoxic state. As IFN gamma (IFNG) signalling promotes cytotoxicity, all observed cytotoxic polarisation will likely be augmented by their upregulation of IFNG (NK-a) or its receptor IFNGR1 (NK-c and T8-c). Notable upregulation of C-C Motif Chemokine Ligands (CCL) known to govern immune cell chemotaxis are observed for all macrophage polarisations, with a conserved CCR2 axis (CCL2, CCL7 and CCL8) for Mac-a, Mac-c and Mac-e. The Mac-c and Mac-e polarisations present with upregulated M2 macrophage marker (MRC1 (CD206) or CD209) expression, implicating them as M2-like macrophages. Conversely, Mac-a presents with upregulated expression of pro-inflammatory CXCL9 and CXCL10 cytokines, indicative of M1-like macrophages. IL-1 and GM-CSF-induced Mono-c and Mono-e polarisations upregulate M1 macrophage differentiation marker VCAN, suggestive of monocyte to M1-like macrophage differentiation. Both IL-1-induced Mono-c and IL-1 beta-induced Mac-c polarisations have upregulated monocyte/macrophage migration (JAML) and differentiation (MAFB) marker expression.

To conclude, our in silico modelling predicts that the ZIKV-induced brain tumour cell secretome would induce a diverse response in non-tumour-resident immune cells by endocrine signalling. We predict this response to include (i) effector memory CD4 + T cells (T4-a and T4-c), (ii) cytotoxic CD8+ T cells (T8-c), (iii) cytotoxic NK cells (NK-a and NK-c), (iv) M1-like (Mac-a) and M2-like (Mac-c and Mac-e) macrophages, (v) M1-like macrophage-destined monocytes (Mono-c and Mono-e), and (vi) neutrophils, cDC and other immune cell polarisations of currently undefined phenotype.

## Discussion

In the current study, we perform the first multi-omics-based investigation of ZIKV infection in paediatric brain tumour cells. At DEG, biological process and pathway levels, we demonstrate a stark contrast in the response of ZIKV-infected brain tumour cells to that of CZS patient-derived NPCs. ZIKV infection leads to a potent anti-tumoural immune response against mouse glioma, resulting in tumour clearance and long-term immunity against tumour cells^33–35^. It is currently unknown what ICD mechanisms orchestrate this crucial efficacious response. Here, our multi-omics analysis highlighted the involvement of TNF and cytokine signalling as indicators of ICD in ZIKV-infected childhood brain tumour cells^42^. Previous observations by members of our research team support these observations and have shown that (i) this upregulated transcriptomic TNF and cytokine response is also observed in ZIKV-infected neuroblastoma cells, (ii) ZIKV-infected brain organoids co-cultured with USP7 or USP13 cells upregulate expression of TNF and a limited number of related cytokines, and (iii) ZIKV-infected canine glioblastoma cells secrete elevated levels of interleukins^31,32,36^.

TNF signalling is a diverse signalling pathway capable of regulating cell proliferation, cell death and immune responses^43^. Contrasting studies report TNF-alpha as anti-tumoural (cytotoxic or cytostatic) or pro-tumoural for medulloblastoma, and that both TNF-alpha transgene and TNFR antagonist treatment portray favourable outcomes in xenograft models^44–46^. Regarding OVs, two independent groups working on the oncolytic myxoma virus report contrasting results that either TNF-alpha transgene or blockade enhances OV efficacy^47,48^. Thus, the clinical utility of TNF-alpha in paediatric brain tumour therapy and OV therapy requires further delineation. Here, we observe recombinant TNF-alpha as pro-tumoural rather than cytotoxic to medulloblastoma and ATRT cells under normal conditions. Additionally, we identify TNF-alpha as a marker of poor prognosis in medulloblastoma, likely in part due to enhanced tumour growth^43^. Interestingly, recombinant TNF-alpha reduces cell viability of ZIKV-infected brain tumour cells, suggesting that in the context of oZIKV therapy, TNF-alpha is anti-tumoural as it enhances brain tumour cell oncolysis by ZIKV. This response is TNFR1-dependent, as TNFR2 activation requires membrane-bound TNF-alpha^49^. While we did not observe TNF-alpha as anti- or pro-viral in medulloblastoma or ATRT cells, it is antiviral in ZIKV-infected adult glioblastoma cells^50^. Our in silico analysis indicates that all medulloblastoma TME immune cells are primed to respond to TNF-alpha paracrine signalling and that TNF-alpha endocrine signalling would polarise myeloid cells to upregulate pro-survival and innate immune recognition genes. Our observed anti-tumoural role of TNF-alpha during ZIKV oncolysis, its reported antiviral property, and our in silico prediction of its involvement in anti-tumoural immune responses highlight that oZIKV infection could likely be augmented by adjuvant therapy targeting TNF-alpha. This concept is exemplified by oncolytic HSV-1 inducing TNF-alpha-mediated glioma cell death, with transient TNF-alpha blockade being able to enhance both viral replication and mouse survival^51^.

Pro-inflammatory cytokine secretion is a hallmark signature of ICD, and here, we demonstrate by multiplex ELISA that ZIKV infection induces a diverse pro-inflammatory secretome from paediatric brain tumour cells. This natural induction of a diverse pro-inflammatory secretome is essential to oZIKV therapy efficacy because ZIKV’s limited RNA genome size is a barrier to enhancing this response by stable transgene modification. Currently, only a limited number of multiplex ELISA assays have assessed the secretome of ZIKV-infected brain tumour cells^29,36^. A 25-plex ELISA, measuring 23 of the 34 cytokines we consider here, showed that ZIKV infection only significantly increased CXCL10 and CCL5 secretion and decreased CCL2 secretion from adult glioblastoma cells^29^. The size and diversity of this cytokine response is much smaller than that we show for ZIKV-infected paediatric medulloblastoma and ATRT cells, suggesting that oZIKV infection is more immunogenic for paediatric rather than adult brain tumour cells.

Here, we observe ZIKV infection to elevate secretion of the principal triad of pro-inflammatory cytokines (IL-1, IL-6, and TNF-alpha) by both brain tumour cell lines, in addition to many other cytokines that we report for the first time to be a component of oZIKV infection. Corroborating our findings, three key pro-inflammatory cytokines with known anti-tumoural functions (IL-6, CCL5 and CXCL10) are also significantly secreted by ZIKV-infected glioblastoma cells^29,36^. We observe ZIKV to upregulate secretion of numerous drivers of OV-induced anti-tumoural immune responses, including but not limited to IFN alpha, IL-1, IL-6, CCL5, CXCL8, CXCL10, CXCL12, GM-CSF and TNF-alpha; as reviewed in^52^. In addition to detecting the secretion of numerous cytokines that are OV transgenes or immunotherapy target candidates (Fig. 6A), we observe ZIKV-infected USP13 cells to specifically upregulate IFN alpha and GM-CSF secretion, both of which are FDA-approved cytokine therapies^53^. IFN alpha is a monotherapy for treating various adult cancers and GM-CSF is an adjuvant to high-risk neuroblastoma anti-GD2 immunotherapy^53–55^. Collectively, we show that ZIKV infection induces a clinically diverse pro-inflammatory and anti-tumoural brain tumour secretome primarily composed of cytokines currently employed as OV transgenes, immunotherapy targets or cytokine therapies.

A crucial element of oZIKV efficacy is the ability to heat up the immunosuppressive TIME by paracrine signalling to co-opt tumour-resident immune cell activity and endocrine signalling to orchestrate immune cell infiltration. ZIKV-induced brain tumour immune cell infiltration includes CD4+ T cells, CD8+ T cells, NK cells, monocytes, macrophages, DCs and microglia^33–35^. Modelling these in silico, we predict distinct polarisation states of the majority of these infiltrating immune cells in response to oZIKV infection. Both CD8+ T cells and NK cells infiltrate ZIKV-infected mouse glioma, and here, our modelling indicates anti-tumoural cytotoxic CD8+ T and NK cell polarisations in response to ZIKV-induced brain tumour cell secretome endocrine signalling^33^. This anti-tumoural phenotype is characterised by upregulated expression of the pro-apoptotic granzyme B, which is a principal component of cytotoxic granules, in addition to key markers of IFN gamma signalling, which orchestrate interferon-mediated anti-tumoural immune responses^56^. ZIKV-induced tumour clearance is dependent on cytotoxic CD8+ T cells, and our modelling predicts that this cytotoxic polarisation is in response to IL-1^33^.

Tumour-associated macrophages (TAMs) generally exist in the pro-tumoural and immunosuppressive M2-like state and commonly undergo a phenotypic shift to a pro-inflammatory and anti-tumoural M1-like state following OV infection^57^. M1-like macrophage infiltration has been reported following oZIKV infection of mouse and canine brain tumours in vivo, however, the mechanisms that orchestrate this response are unknown^33,36^. Of the 13 significantly secreted cytokines from ZIKV-infected USP13 medulloblastoma cells, nine (TNF-alpha, GM-CSF, IL-1 beta, IL-6, CXCL1, CXCL10 and CCL3-5) are known to induce the M1-like phenotype, whilst only CCL5 and high levels of IL-1RA may lead to the M2-like phenotype^58^. Here, we observe high TNF-alpha and GM-CSF receptor expression and significant enrichment of their respective pathways in medulloblastoma TAMs. As such, we propose that in response to ZIKV-induced secretion of either TNF-alpha or GM-CSF, these medulloblastoma TAMs would adopt a pro-inflammatory and anti-tumoural phenotype. Utilising the Immune Dictionary, we identify that macrophages are stimulated by IFN alpha and monocytes are stimulated by both IL-1 and GM-CSF to express M1-like macrophage gene markers. In contrast, IL-1 and IL-4 stimulate M2 gene markers in macrophages, which supports the known immunosuppressive effects of these cytokines on TAMs^59^. Therefore, our modelling indicates a predominantly M1-like macrophage phenotype following oZIKV infection with potential for some M2-like macrophage populations.

Our modelling examined the individual interactions between a single cytokine and a single immune cell type. This poses a limitation as it only assesses the indirect effects of ZIKV infection and does not consider other aspects of the highly complex interplay between OVs, tumours, and the immune system, such as direct infection of immune cells by ZIKV. In non-human primates, ZIKV infects immune cells in the lymph nodes, including DCs, macrophages, and B cells^60^. Consequently, further research is warranted to dissect these multifaceted interactions between ZIKV, brain tumours, and the immune system.

To conclude, we investigate ICD signatures following oZIKV infection of paediatric brain tumour cells and propose TNF-alpha as a potential prognostic marker for brain tumour oZIKV therapy, with the only other known prognostic marker being CD24 for neuroblastoma oZIKV therapy^61^. We observe a clinically relevant pro-inflammatory and anti-tumoural ZIKV-induced brain tumour secretome. Through in silico modelling, we predict ZIKV secretome paracrine signalling to remodel TME immune cells and endocrine signalling to drive diverse immune cell polarisations. We propose that this would collectively lead to a pro-inflammatory and anti-tumoural immune response that would heat up the paediatric brain TME and be fundamental to the efficacy of oZIKV therapy. The research we perform here contributes to understanding the molecular mechanisms governing oZIKV infection and the cytokine response which orchestrates the anti-tumoural immune response. This work contributes to the growing body of research indicating the clinical utility of ZIKV as an oncolytic virotherapy for nervous system tumours and will help pave the way towards its application in clinical trials.

## Methods

### Cell and viral culture

Paediatric USP7 ATRT and USP13 medulloblastoma cells were cultured as previously described^24,62^, as were the paediatric CZS-affected patient-derived NPCs (NPC-763-1 and NPC-788-1)^40^. Vero cells were cultured in complete DMEM (Gibco, 41966029) with 10% FBS and 1% Penicillin-Streptomycin (5% CO_2_, 37 °C) and passaged 1:12 every 72 h. All cell lines were regularly tested and confirmed to be free of Mycoplasma. Stocks of two highly conserved Brazilian epidemic ZIKV strains, BeH819966 (KU365779.1) and Paraiba_01 (KX280026.1), were maintained at Instituto Butantan (University of São Paulo) and The Pirbright Institute, respectively. To maintain stocks, Vero cells in serum-free DMEM were infected for 90 min. At 96 hpi or when sufficient CPE was detected, virus was collected, clarified, filtered (0.22 μm) and stored as single use aliquots at -80 °C. Virus was titrated in technical duplicate in Vero cells by plaque forming units (PFU) assay, using a 10-fold dilution series in serum-free DMEM. Following 90 min infection, cells were washed with PBS, a solid overlay of UltraPure 1% LMP agarose (Invitrogen, 16520-100) containing 0.83% complete DMEM was added, and plates were incubated for 72 h. Plaques were fixed with formalin overnight, washed with PBS, and stained with crystal violet for 5 min. All brain tumour cell and NPC infection experiments were performed at a multiplicity of infection (MOI) of 2 for 60 min. Infection experiments for confocal microscopy and RNA-Seq used BeH819966, whilst all remaining infection experiments used Paraiba_01.

### Confocal microscopy

Brain tumour cells were plated on coverslips for 24 h prior to infection, infected and then fixed with 4% PFA for 40 min. Cells were permeabilised and blocked with PBSAT for 30 min, incubated in primary antibody anti-NS2B (Genetex, GTX133308) for 90 min, and then secondary antibody (ThermoFisher, A-11007) for 60 min. Cells mounted in VECTASHIELD Medium with DAPI (Vector Laboratories, H-1200-10). Coverslips were washed with PBS three times between incubations. Coverslips were stored until imaged via confocal microscopy with ZEN Software.

### Bulk RNA-sequencing

At 12, 18 and 24 hpi, cells were collected, washed with PBS and stored as pellets at -80 °C (*N* = 3). High-quality total RNA was purified using the Monarch Total RNA Miniprep Kit (NEB #T2010S) per the manufacturer’s protocol. Bulk mRNA sequencing was performed by Novogene (UK) Company Limited using the Illumina NovaSeq 6000 system (≥ 20 million 150 bp paired-end reads per sample). The RNA-Seq data processing pipeline consisted of FastQC (V0.11.9-0), Trim Galore (V0.6.6-0), HISAT2 (V2.2.0), Samtools (V1.11) and Subread (V2.0.1). Reads were aligned against the Homo Sapien GRCh38 and ZIKV BeH819966 genome, and differential gene expression analysis was performed using DESeq2 to identify DEGs in the ZIKV-infected brain tumour (padj ≤ 0.05, Fold Change ≥ 0) and NPCs (padj ≤ 0.05, Fold Change ≥ 1.5) at given time points of infection relative to uninfected Mock samples. Upregulated and downregulated DEGs were submitted to DAVID to identify upregulated and downregulated GO Biological Processes, KEGG pathways and Reactome pathways, respectively^63^. Significance values were corrected for multiple testing using the Benjamini and Hochberg method (padj ≤ 0.05). Hierarchical clustering ordered the terms (rows) by similarity across the twelve infection conditions. The top 120 DEGs at 24 hpi were determined by ranked absolute Log2(Fold Change) (LFC) value, and KEGG pathway analysis was performed for these brain tumour DEGs using DAVID. The publicly available RNA-Seq datasets of ZIKV-infected tumour cells were sourced from GEO2R with the GEO accessions GSE114907 (4121 GSC, MOI 1, 48 hpi, *N* = 3), GSE102924 (387, 3565 and 4121 GSC, MOI 5, 48 hpi, *N* = 3) and GSE149775 (SH-SY5Y, MOI 5, 24 hpi, *N* = 3)^27,29,64^. All DEGs are presented as LFC with p-values corrected for multiple testing by the Benjamini and Hochberg method (padj ≤ 0.05), with infected samples relative to uninfected Mock samples.

### Data availability

The RNA-Seq data generated in the present study have been deposited to the NCBI Gene Expression Omnibus (GEO) with the GEO accession GSE277900.

### Recombinant TNF assays

USP7 and USP13 cells were treated with 100 µg/ml recombinant TNF-alpha (Peprotech, 300–01 A), TNFSF9 (Peprotech, 310 − 11), TNFRSF9 (Peprotech, 310 − 15) or a 0.1% BSA vehicle control. For Incucyte Live Cell Growth Analysis, USP7 and USP13 cells were plated at low confluence in sufficient media and allowed to grow until the first condition of each cell line reached 95% confluency. Confluence was determined by the Confluence Basic Analyser, with the phase map trained using AI specifically off each cell line (label-free) at varying confluency to ensure the accuracy of readings during live cell imaging. Cell culture supernatant was collected at 48 hpi for infection experiments, clarified, stored as single-use aliquots at -80 °C, and titrated via PFU in Vero cells. Cell viability was determined using the CellTiter-Glo Assay (Promega, G7572), per the manufacturer’s protocol. For all recombinant TNF assays, significance was defined and corrected for multiple testing by two-way ANOVA with Dunnett’s multiple correction (padj ≤ 0.05).

### Medulloblastoma patient survival analysis

Medulloblastoma patient gene expression and survival data were sourced using GEO2R for GEO accession GSE85218 (GPL22286)^65^. ATRT patient survival analysis could not be performed due to the extreme scarcity of ATRT datasets. Patient survival was censored at five years, and Kaplan-Meier survival analysis was performed for the remaining 375 patients. Logrank P value was determined by univariate Cox regression (*p* ≤ 0.05), comparing survival between the upper and lower quartiles based on candidate gene expression. Hazard rate (HR) with 95% confidence intervals are reported. Analysis was performed using KMplot^66^.

### ProcartaPlex multiplex ELISA assay

At 12, 24 and 48 hpi, cell culture supernatant was collected from infected USP7 and USP13 cells, centrifuged, and stored as single-use aliquots at -80 °C (*N* = 3, *n* = 2). ProcartaPlex kits quantified supernatant protein concentration of 34 cytokines (Invitrogen, EPXR340-12167-901) and 15 immune checkpoint proteins (Invitrogen, EPX010-15901-901 and EPX14A-15803-901), as per the manufacturer’s protocol. Data was collected using a Bio-Plex 200 and processed on the ThermoFisher ProcartaPlex Analysis App. Optimised standard curves were plotted with a 5 PL Logistic fit. For each condition, outliers were omitted and technical duplicates were averaged. Protein concentrations which were above the limit of detection but below the standard curve lower limit of quantification (LLOQ) were included in Figs. 4C–E and 5B, as these protein concentrations were deemed to be more informative than plotting missing values. The LLOQ for each specific analyte is plotted so protein concentrations with a lower confidence level are apparent. Multiple unpaired t-tests defined significance with Benjamini and Hochberg correction (FDR ≤ 0.05). PCA was performed on the averaged Net MFI values.

### Paracrine signalling analysis

A scRNA-Seq log-transformed normalised gene counts matrix of 28 untreated primary paediatric human medulloblastoma tumours was sourced from the Tumor Immune Single-cell Hub 2 (TISCH2)^67^. Analysis was limited to medulloblastoma due to the absence of ATRT scRNA-Seq datasets. The scRNA-Seq dataset consisted of 37,445 cells: 32,926 Tumour cells, 2665 monocytes/macrophages, 1590 CD8+ T cells and 264 neutrophils (GEO accession GSE155446)^68^. TISCH2 processed and analysed the dataset using the MAESTRO workflow, and the gene counts matrix reports the average expression per cell type. GSEA and the expression of specific cytokine receptors of interest were plotted using the log-transformed normalised gene counts matrix. GSEA was performed by comparing each immune cell type against the tumour cells for immune cell pathway enrichment, and then tumour cells against all immune cells for tumour cell pathway enrichment^69^. Analysis was performed with the Reactome database, and significant pathways (*p* ≤ 0.05 and FDR ≤ 0.25) relating to Interleukin-, Interferon-, TNF- and Chemokine-receptor signalling were plotted.

### Endocrine signalling analysis

The Immune Dictionary, created by Cui et al., contains scRNA-Seq data of mouse lymph node immune cells following in vivo treatment with 86 different recombinant cytokines, and includes over 1,400 cytokine–cell type interactions^41^. To promote reproducibility between our study and future studies that incorporate the Immune Dictionary, here, we utilised the analysed scRNA-Seq data available through the Immune Dictionary Application (IDA)^41^. From this rich resource, we sourced data for the six ZIKV-infected USP7 or USP13 cell-secreted cytokines that Cui et al. show to drive polarisation states of the main anti-tumoural immune response-related lymphoid (CD4+ T, CD8+ T and NK cells) and myeloid (monocytes, macrophages, DCs and neutrophils) cell types. These cytokines are IFN alpha, IL-1 alpha, IL-1 beta, IL-4, TNF-alpha and GM-CSF, and this approach highlighted 27 cytokine-cell interactions of interest. The scRNA-Seq data was analysed in the IDA via a two-sided Wilcoxon rank-sum test comparing normalised gene expression values of cytokine treatment versus PBS control (*N* = 3), with significance corrected for multiple testing (FDR ≤ 0.05). For each cytokine-cell interaction, we downloaded the Top 20 upregulated cytokine-induced DEGs from the IDA (Supplementary Table 2). Extensive literature searching identified whether these DEGs were cytokines, cytokine receptors, cytotoxicity-related genes, survival-related genes, cell lineage markers, cytokine signalling regulators, or immune cell activation, migration or differentiation genes. The DEGs which conferred immune cell activity or phenotype were collectively considered for each cytokine-cell interaction to deduce the immune cells’ phenotype following cytokine stimulation. These specific DEGs were mapped from their mouse to human orthologs using g: Profiler for inclusion in Fig. 7^70^.

## Electronic supplementary material

Below is the link to the electronic supplementary material.

Supplementary Material 1

Supplementary Material 2

## Data Availability

The RNA-Seq data generated in the present study have been deposited to the NCBI Gene Expression Omnibus (GEO) with the GEO accession GSE277900.
