# Supplementary material for: Multi-omics analysis reveals key immunogenic signatures induced by oncolytic Zika virus infection of paediatric brain tumour cells
Source: Sci Rep. 2025 Apr 16;15:13090. doi: 10.1038/s41598-025-97804-8 (PMC12003866; doi:10.1038/s41598-025-97804-8)
Supplement: Supplementary file 2 — Supplementary Material 2 [file 41598_2025_97804_MOESM2_ESM.docx]

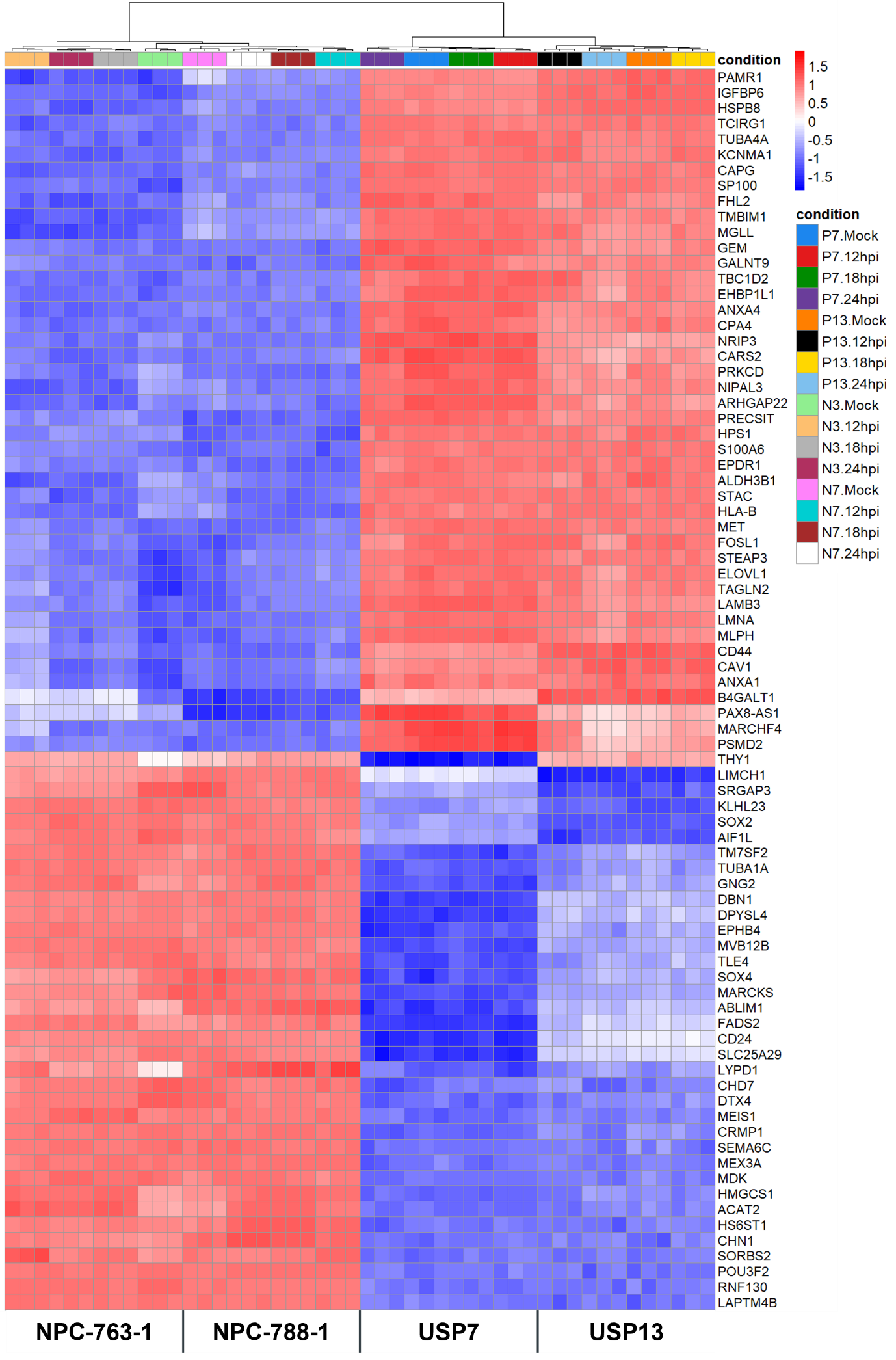


***Supplementary Figure 1. Heatmap of ZIKV-infected RNA-Seq samples.***

*Heatmap of the Top 80 differential genes across all 48 bulk RNA-Seq samples. Hierarchical clustering arranged the heatmap by condition (columns) and genes (rows); the dendrogram for the latter was omitted to aid visualisation. Abbreviations, Zika virus (ZIKV), USP7 (P7), USP13 (P13), NPC-763-1 (N3), NPC-788-1 (N7), hours post-infection (hpi).*


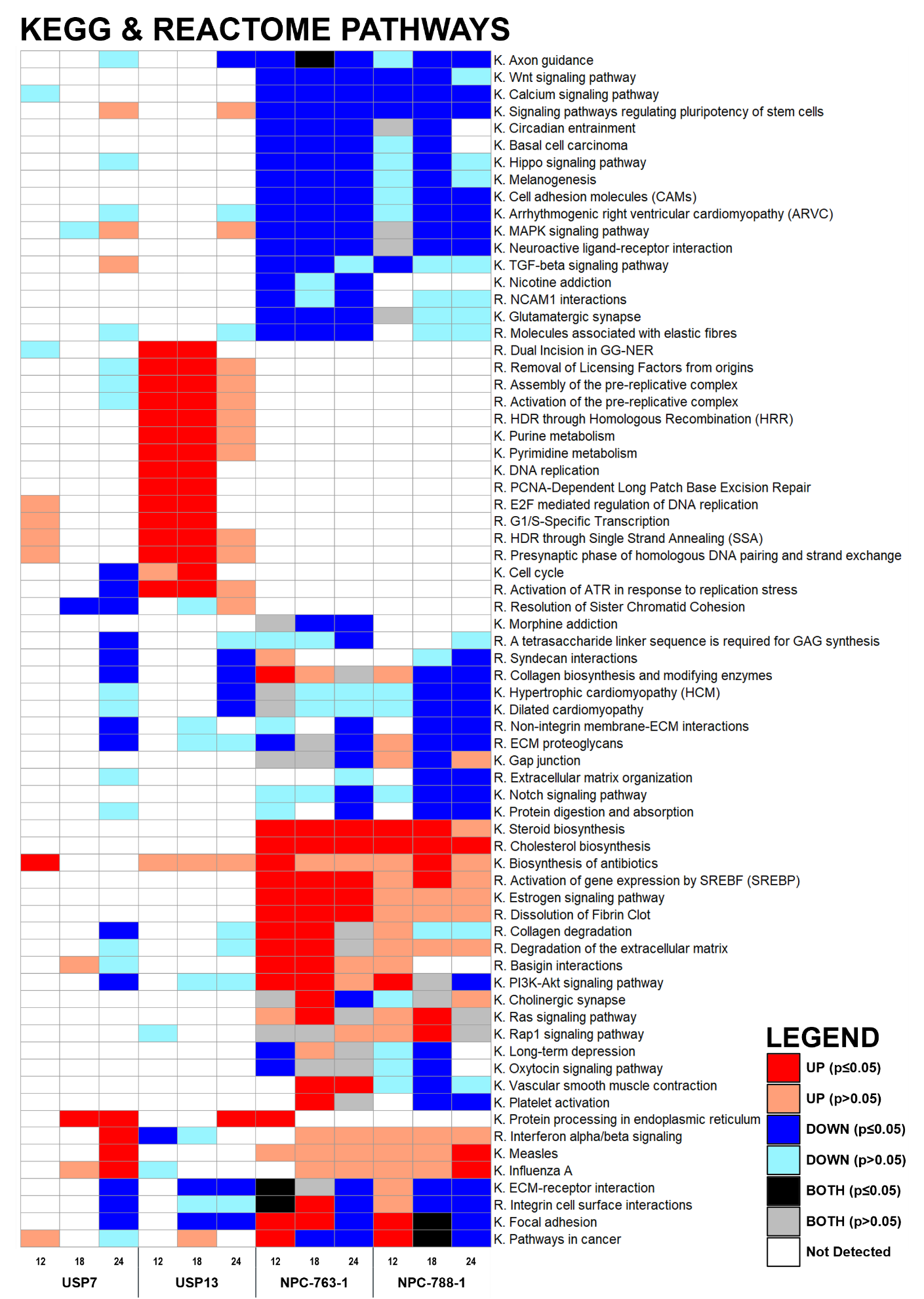


***Supplementary Figure 2. Heatmap of differentially enriched pathways following ZIKV infection.***

*Heatmap of the enriched KEGG (K.) and Reactome (R.) Pathways in the DEG lists at 12, 18 and 24 hpi in brain tumour and neural precursor cells. All terms which were significant (p ≤ 0.05) in at least one of the twelve infection conditions were plotted, and any non-significant (p > 0.05) enrichment of the given term across the remaining conditions was also plotted. If a given term was enriched in both the upregulated and downregulated DEG lists, then it was labelled as BOTH. Significance values are corrected for multiple testing using the Benjamini and Hochberg method (padj ≤ 0.05). Abbreviations, Zika virus (ZIKV), differentially expressed gene (DEG), USP7 (P7), USP13 (P13), NPC-763-1 (N3), NPC-788-1 (N7), KEGG (K.), REACTOME (R.), hours post ZIKV infection (hpi), adjusted p-value (padj).*
